# Cell tip growth underlies injury response of marine macroalgae

**DOI:** 10.1371/journal.pone.0264827

**Published:** 2022-03-17

**Authors:** Maki Shirae-Kurabayashi, Tomoya Edzuka, Masahiro Suzuki, Gohta Goshima

**Affiliations:** 1 Sugashima Marine Biological Laboratory, Graduate School of Science, Nagoya University, Sugashima, Toba, Japan; 2 Kobe University Research Center for Inland Seas, Iwaya, Awaji, Hyogo, Japan; 3 Division of Biological Science, Graduate School of Science, Nagoya University, Furo-cho, Chikusa-ku, Nagoya, Aichi, Japan; Lovely Professional University, INDIA

## Abstract

Regeneration is a widely observed phenomenon by which the integrity of an organism is recovered after damage. To date, studies on the molecular and cellular mechanisms of regeneration have been limited to a handful of model multicellular organisms. Here, the regeneration ability of marine macroalgae (Rhodophyta, Phaeophyceae, Chlorophyta) was systematically surveyed after thallus severing. Live cell imaging on severed thalli uncovered the cellular response to the damage. Three types of responses–budding, rhizoid formation, and/or sporulation–were observed in 25 species among 66 examined, proving the high potential of regeneration of macroalgae. The cellular and nuclear dynamics were monitored during cell repair or rhizoid formation of four phylogenetically diverged species, and the tip growth of the cells near the damaged site was observed as a common response. Nuclear translocation followed tip growth, enabling overall distribution of multinuclei or central positioning of the mononucleus. In contrast, the control of cell cycle events, such as nuclear division and septation, varied in these species. These observations showed that marine macroalgae utilise a variety of regeneration pathways, with some common features. This study also provides a novel methodology of live cell imaging in macroalgae.

## Introduction

Regeneration is widely observed in multicellular organisms; injured tissue (*e*.*g*. the human liver and lizard’s tail) or, in some cases, the whole body of an organism (*e*.*g*. planaria and moss) is eventually recovered through cell proliferation and differentiation [1, 2]. Marine organisms often incur injuries, typically caused by storms or predators. As a result, they have certain response mechanisms to this damage. However, the regeneration process has rarely been described at a cellular level for marine organisms, and the underlying mechanisms remain poorly understood.

Marine macroalgae, or seaweeds, are an important component in marine ecology [3]. Three types of seaweeds, namely, red (Rhodophyta), brown (Phaeophyceae), and green (Chlorophyta) macroalgae, are widely distributed, particularly in coastal areas. Phylogenetically, red and green algae and land plants constitute a single clade, whereas brown algae are more distinct [4]. Their ability to photosynthesise makes them critical CO_2_ consumers and oxygen producers. They also serve as food sources and habitats for benthic and nektonic animals, and the interaction with animals raises the risk of injury. A high potential for regeneration has been described in some macroalgal species. When a filament of *Griffithsia pacifica* Kylin was severed, regrowth of the cell next to the damaged cell was observed [5]. In the red alga *Gracilariopsis chorda* (Holmes) Ohmi, experimentally severed thalli were returned to the ocean, and vegetative regeneration was subsequently observed [6]. Furthermore, in the brown alga *Dictyota dichotoma* (Hudson) J.V. Lamouroux, budding and rhizoid formation from a severed surface occurs in juvenile thalli [7]. *In vitro* regeneration assays have been also conducted, which involved artificially prepared protoplasts or the callus [8, 9]. In these assays, protoplasts of the green algae *Ulva* (sea lettuce) and *Bryopsis* regenerated to form the thallus [10, 11]. Explants of gracilarialean red algae generate calli, which are stimulated by plant growth hormones [12, 13]. However, the use of different assays in different studies makes it difficult to deduce whether there is a common mode of regeneration in macroalgae. Furthermore, how cells initially respond to injury remains largely unclear at both the cellular and intracellular level, as high-resolution time-lapse imaging of the cellular and intracellular components has scarcely been applied.

This study aimed at surveying the injury response of 66 marine macroalgae. The response was monitored at the macroscopic and cellular level in order to identify common features among the phylogenetically diverse species.

## Materials and methods

### Macroalgae collection

Macroalgae were predominantly collected from the intertidal zone of the seashore, in front of the laboratory (Lat. 34°29’8” N, Long. 136°52’32″ E) on the 25^th^ and 26^th^ of March 2020, and on the 30^th^ of March and 1^st^ of April 2021 (the ocean water temperature was ~14°C). Samples were also collected from the outside tank of the laboratory, as well as from the ropes of the floating pier (S1 Fig).

### Identification of macroalgae

Collected macroalgae were examined to determine their probable genera and/or species; monographic publications and floristic studies were used to confirm the morphology and identify the species [14, 15]. The nomenclature used in this study follows Algaebase [16]. DNA barcode sequencing was performed for some specimens to confirm and/or correct the morphological identification. The plastid *rbc*L, mitochondrial cox1, nuclear 18S rDNA, and/or rDNA ITS1-5.8S-ITS2 region were amplified and sequenced. Genomic DNA was extracted using the DNeasy Plant Mini Kit (Qiagen) from field-collected specimens dried by silica gel. Total DNA was used as the template for polymerase chain reactions (PCR) in which the *rbc*L, cox1, 18S rDNA, and/or rDNA ITS1-5.8-ITS2 loci were amplified using KOD-ONE (Toyobo Co. Ltd., Osaka, Japan) and 2720 PCR Thermal Cycler (Applied Biosystems, Foster City, CA, USA). In some cases, the squeezed extract or homogenised thallus was used as the PCR template. The primers used for PCR amplification are shown in Table 1. The temperature cycling protocol was as follows: 2 min at 94°C for an initial denaturation step, 35 cycles of 15 s denaturation at 94°C, 30 s primer annealing at 46°C, and 1 min extension at 68°C. The amplified DNA fragments were purified by ethanol precipitation and sequenced by eurofins genomics (Luxembourg). Reverse and direct chromatograms were assembled using ClustalW software. The sequences were registered at DNA Data Bank of Japan (DDBJ) and the accession numbers are listed in S1 Table.

**Table 1:** Primers used for PCR amplification in this study.

Locus	Primer ID	Primer sequences	Reference
*rbc*L	981_ rbcL_Bryopsis-F1	CACCTGATTACCAAGTAAAAGATAC	Designed in this study
	982_ rbcL_Bryopsis-R1	GGCTGCTAATTCAGGACTCCA	Designed in this study
	003_Dasya_rbcL_F1	AAAACATTCCAAGGTCCTGCAAC	Designed in this study
	006_Dasya_rbcL_R2	TCTTTCCACAGGTCTAATGCTGTTTGT	Designed in this study
	011_rbcL_F8	GGYGTAATTCCATATGCWAAAATG	Modified from [17]
	012_rbcL_Rh3	TYAAYTCTCARCCDTTYATACG	Modified from [18]
	013_rbcL_R1150	GCATTTGWCCACARTGAATACC	Modified from [17]
	014_rbcL_R1381	ATCTTTCCATAAATCTARAGC	Modified from [17]
*cox*1	GazF2	CCAACCAYAAAGATATWGGTAC	[19]
	GazR2	GGATGACCAAARAACCAAA	[19]
18SrDNA	G01	CACCTGGTTGATCCTGCCAG	[20]
	G07	AGCTTGATCCTTCTGCAGGTTCACCTAC	[20]
	007_Dasya_18SrDNA_F1	ACGGTATCTGATCGTCTTCGATCC	Designed in this study
	009_Dasya_18SrDNA_R1	AGGTTCACCTACGGAAACCTTGT	Designed in this study
rDNA ITS	015_Ulva_ITS1-U2	GTGGGTGATCTGGAAACCCTGGAGG	[21]
	016_Ulva_ITS5-m	CCCATACCGGCACCGGTACC	[21]
	017_Ulva_ITS4-U	CGCCGYTACTARGGGAATCC	[21]
	ITS1_Bryopsis-F	AGGAGAAGTCGTAACAAGGT	Designed in this study
	ITS4_Bryopsis-R	TCCTCCGCTTATTGATATGC	Designed in this study

### Severing experiments

Most of the macroalgae were severed within a week after collection at room temperature (~15°C), as detailed below. The samples for which severing was not conducted instantly were kept in the tanks filled with 8 litres of natural seawater at 15°C and then acclimatised to room temperature for a day before severing. For *Codium fragile* (Suringar) Hariot, medullary filaments were isolated and grown for ≥ 2 weeks at room temperature, followed by severing. The thalli of each species were washed with filtered seawater and severed in approximately 5 mm (× 1–5 mm) fragments using surgical knives (Surgical blade, No. 15 and No. 23, Feather, Japan) in 8-cm petri dishes. The surgical knives were sterilised with 70% ethanol prior to each experiment. Five to ten fragments from a single thallus were immersed in approximately 50 ml modified seawater (MSW) in the petri dishes for long-term culture, which was prepared using the following method: ocean surface water (salt concentration 3.0–3.4%) was filtered using a 0.45-μm Millipore Stericup, autoclaved, and supplied with GeO_2_ (final concentration, 1 mg/L) and Provasoli’s enriched seawater (PES medium [22]) or Daigo’s IMK medium (FUJIFILM, Tokyo, Japan). The samples were cultured at room temperature with constant light exposure (1200 lux, 13 μ mol / m^2^ s) without water flow. The severed site of the algae was checked with a stereomicroscope every day for a maximum of 21 days. Twenty two species were collected during the springtime of two successive years and the experiments were repeated at least twice; for them, reproducible response (or no response) to severing was confirmed (Table 2, asterisks). In each severing experiment, a portion of the thalli was kept unsevered under the same culture conditions to make sure that they were alive during the observation period (21 days). For raw sections of *Gelidium elegans* Kützing buds, the thalli were manually severed with a razor (FAS-10, Feather, Japan). For frozen sections of this species, the served thalli were fixed with 4% paraformaldehyde in PHEM buffer (60 mM Pipes, 25 mM Hepes, 10 mM EGTA, 2 mM MgCl_2_, pH 6.9) and frozen with OCT medium at -80°C for 1 h. Sections were prepared using CM3000 Cryostat (Leica biosystems, Wetzlar, Germany).

**Table 2:** Summary of the response to thallus severing.

Species name	Fomation			Species name	Fomation			Species name	Fomation			Species name	Fomation		
	Bud	Rizhoid	Spore		Bud	Rizhoid	Spore		Bud	Rizhoid	Spore		Bud	Rizhoid	Spore
*Cladosiphon umezakii* or *Tinocladia crassa*	* *	* *	* *	*Antithamnion* sp	* *	* *	* *	*Gracilariopsis chorda*	*✔*▯**	* *	* *	*Bryopsis* sp.*	*✔*▯**	*✔*▯**	* *
Colpomenia sinuosa*	* *	* *	* *	*Asparagopsis taxiformis*	*✔*▯**	*✔*▯**	* *	*Grateloupia crispata*		* *	* *	Blidingia minima*	* *	*✔*▯**	*✔*▯**
*Dactylosiphon bullosus*	* *	* *	* *	*Binghamia californica*	* *	* *	* *	*Grateloupia imbricata*	* *	* *	* *	Cladophora albida*	*✔*▯**	*✔*▯**	*✔*▯**
Dictyopteris undulata*	* *	* *	* *	*Ceramium* sp.*	*✔*▯**	*✔*▯**	* *	*Grateloupia livida*	*✔*▯**	* *	* *	*Codium coactum*	*✔*▯**		* *
*Dictyota dichotoma*		* *	* *	Champia parvula*	* *	* *	* *	*Grateloupia* sp.	* *	* *	* *	Codium fragile *	*✔*▯**		* *
*Feldmannia mitchelliae*	*✔*▯**	*✔*▯**	* *	*Chondracanthus chamissoi*	*✔*▯**	* *	* *	*Laurencia* sp.*	* *	* *	* *	*Derbesia* sp.	*✔*▯**	*✔*▯**	* *
Hydroclathrus clathratus*	* *	* *	* *	*Chondracanthus okamurae*	* *	* *	* *	*Gymnogongrus flabelliformis*	* *	* *	* *	*Monostroma nitidum*	* *	* *	* *
*Ishige foliacea*	* *		* *	*Chondria crassicaulis*	* *	* *	* *	*Lomentaria hakodatensis*	* *	* *	* *	Ulva australis*	* *	* *	*✔*▯**
*Myelophycus simplex*	* *	*✔*▯**	* *	Colaconema codicola*	*✔*▯**	*✔*▯**	* *	Martensia jejuensis*	*✔*▯**	* *	* *	*Ulva linza*	* *	* *	*✔*▯**
Padina arborescens*	* *	* *	* *	*Chondrus ocellatus*	* *	* *	* *	*Melanothamnus japonicus*	*✔*▯**	*✔*▯**	* *	*Ulva onoi*	* *	* *	* *
*Sargassum fulvellum* or Sargassum muticum	* *	* *	* *	*Corallina maxima*	* *	*✔*▯**	* *	*Pachymeniopsis sp*.		* *	* *	* *	* *	* *	* *
Sargassum fusiforme*	* *	* *	* *	*Dasya* sp.*	*✔*▯**	*✔*▯**	* *	*Pterocladiella tenuis*	*✔*▯**	* *	* *	* *	* *	* *	* *
Sargassum giganteifolium	* *	* *	* *	*Dasysiphonia Japonica*	* *	* *	* *	*Pterothamnion* sp.	*✔*▯**	* *	* *	* *	* *	* *	* *
Sargassum horneri*	* *	* *	* *	*Dudresnaya japonica*	* *	* *	* *	Schizymenia dubyi*	* *	* *	* *	* *	* *	* *	* *
*Sargassum macrocarpum*	* *	* *	* *	*Fushitsunagia catenata*	*✔*▯**	* *	* *	*Scinaia okamurae*	* *	* *	* *	* *	* *	* *	* *
Sargassum muticum	* *	* *	* *	*Gelidium elegans*	*✔*▯**	* *	* *	*Symphyocladia linearis*	*✔*▯**	*✔*▯**	* *	* *	* *	* *	* *
*Sargassum patens*	* *	* *	* *	Gigartinales Rhizophyllidaceae*		* *	* *	Tsengia lancifolia*	* *	* *	* *	* *	* *	* *	* *
*Sargassum thunbergii*	* *	* *	* *	Gracilaria textorii*	* *	* *	* *	*Wrangelia tanegana*	* *	*✔*▯**	* *	* *	* *	* *	* *
*Sargassum yamamotoi*	* *	* *	* *	* *	* *	* *	* *	* *	* *	* *	* *	* *	* *	* *	* *
Scytosiphon lomentaria*	* *	* *	* *	* *	* *	* *	* *	* *	* *	* *	* *	* *	* *	* *	* *
*Spatoglossum crassum*	* *	* *	* *	* *	* *	* *	* *	* *	* *	* *	* *	* *	* *	* *	* *
Undaria pinnatifida*	* *	* *	* *	* *	* *	* *	* *	* *	* *	* *	* *	* *	* *	* *	* *

Grey: untested. Asterisk: cut experiments were repeated at least twice.

### Imaging

Stereomicroscopic images were acquired using an ILCE-QX1 camera (Sony, Tokyo, Japan) attached to SMZ800N microscope (Nikon). A Plan 1× lens was used. Section imaging was acquired with an ILCE-QX1 camera (Sony) attached to ECLIPSE E200 (Nikon). For time-lapse microscopy, thalli were severed into < 2 mm pieces in the MSW and treated with Hoechst 33342 at 10 μg/mL (final) for DNA and cell wall staining. After 30 min incubation with the Hoechst dye, the thalli were injected into the microfluidic device which has previously been used for moss imaging [23, 24]. In brief, a polydimethylsiloxane (PDMS) device was attached to a glass-bottom dish (dish diameter 35 mm; glass diameter 27 mm; glass thickness 0.16–0.19 mm), into which severed thalli were injected by needle (S2 Fig). The height of the device was 15 μm. Hoechst imaging was performed at 23°C with a Nikon inverted microscope (Ti2) attached to a 40× lens (0.95 NA), a 405-nm laser, a CSU-10 spinning-disc confocal unit, and a CMOS camera (Zyla, Andor). Depending on the species under observation, sequential image acquisition time intervals varied between 10 and 30 min. The microscopes were controlled using NIS Elements software (Nikon). The data were analysed using FIJI.

Four species were subjected to live cell imaging with spinning-disc confocal microscopy, namely, *Colaconema codicola* (Børgesen) Stegenga, J.J. Bolton & R.J. Anderson (red), *Dasya* sp. (red), *Cladophora albida* (Nees) Kützing (green), and *Codium fragile* (green). These specimens were obtained during the springtime of two successive years and were kept in the laboratory tank at room temperature for >1 year. Therefore, experiments were conducted repetitively.

## Results

### Budding, rhizoid formation, and sporulation are three major injury responses

Among the macroalgal thalli collected in front of the Sugashima marine laboratory (S1 Fig), 54 species were identified through visual inspection (26 red, 21 brown, and 7 green algae). In addition, 14 species that could not be morphologically identified were collected (Table 2). Consequently, DNA barcode sequences were determined for these unidentified species and compared with those registered in the database (S1 Table). Two species died before the experiment, so serving experiments were conducted on 66 species.

**Fig 1:**
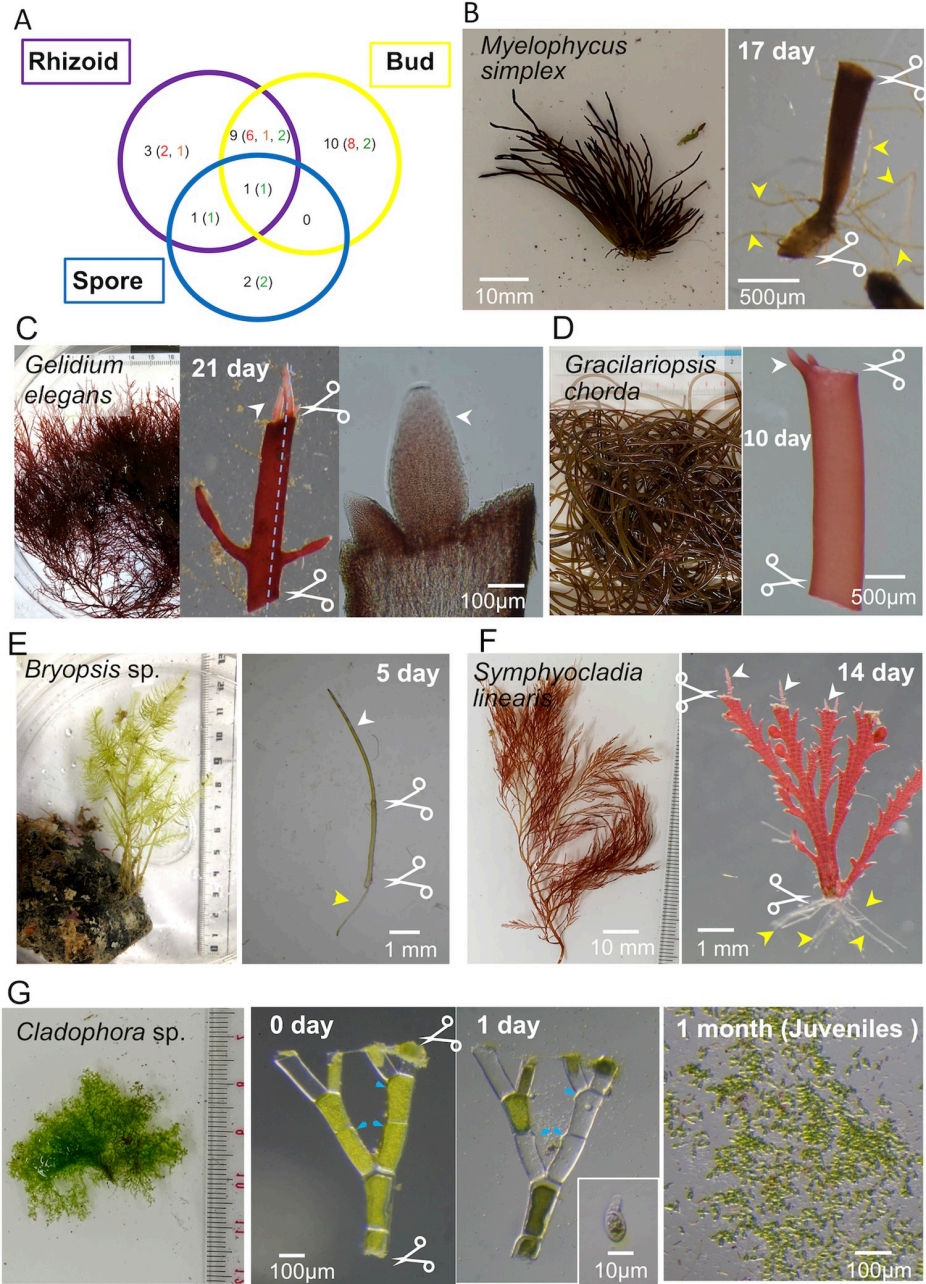
Budding, rhizoid formation, and sporulation are the major responses to injury. (A) Number of macroalgal species that showed the indicated response. The numbers of red, brown, and green algae are indicated by their colours. (B) Rhizoid formation at the basal cut site in *Myelophycus simplex* (Harvey) Papenfuss. (C) Budding at the cut site of *Gelidium elegans*. The rightmost panel is a section image of the blue dotted line in the second panel. Buds appeared in the inner part of the cut surface. (D) Buds appeared at the edge of the cut surface in *Gracilariopsis chorda* (Holmes) Ohmi. (E) Bud and rhizoid formation after cutting of *Bryopsis* sp. (F) Bud and rhizoid formation after cutting of *Symphyocladia linearis* (Okamura) Falkenberg. (G) Sporulation in *Cladophora albida*. Within 24 h after injury, spores were formed and released from the thalli. White arrowhead, bud; yellow arrowhead, rhizoid; blue arrowhead, pore through which spores are released.

Results from the 21 day-follow up of the severed sites in the algae are summarised in Fig 1A and Table 2. Immediate death (withering and/or loss of their original colour) or no specific response was observed for 41 species following severing, whereas the other 25 species showed three major responses: rhizoid formation (yellow arrowheads in Figs 1B, 1E, 1F, S3, S6 and S7), budding (white arrowheads in Figs 1C–1F and S3), and sporulation (Figs 1G and S7). Two or three of these responses were simultaneously observed in 11 species (Figs 1E, 1F and S3). Even though many specimens were severed in multiple locations, no site-dependent responses were observed.

There was a clear tendency in the response type for each algal lineage. The majority of the brown algae showed no response (2/22 formed buds and/or rhizoids) (Figs 1A and S8). In contrast, 47% (16) of the red algae specimens showed budding and/or rhizoid formation. Sporulation was only observed in green algae (4/10 showed sporulation; among them, the ability of *Ulva linza* to sporulate is consistent with a previous study [25]). In all cases, buds and rhizoids were formed at the apical and basal regions, respectively, indicating that the apicobasal polarity was maintained after being severed (*e*.*g*. Fig 1F). Depending on the species, buds were observed on the inner part of the severed surface (Figs 1C and S9) or at its edge (Fig 1D). During regeneration of buds or rhizoids, callus-like cell masses were never observed. Overall, marine macroalgae have a high regeneration potential, that is limited to three patterns, budding, rhizoid formation, and sporulation.

### Regeneration in uninuclear *Colaconema codicola* is characterised by cell tip growth and nuclear division, followed by septation

**Fig 2:**
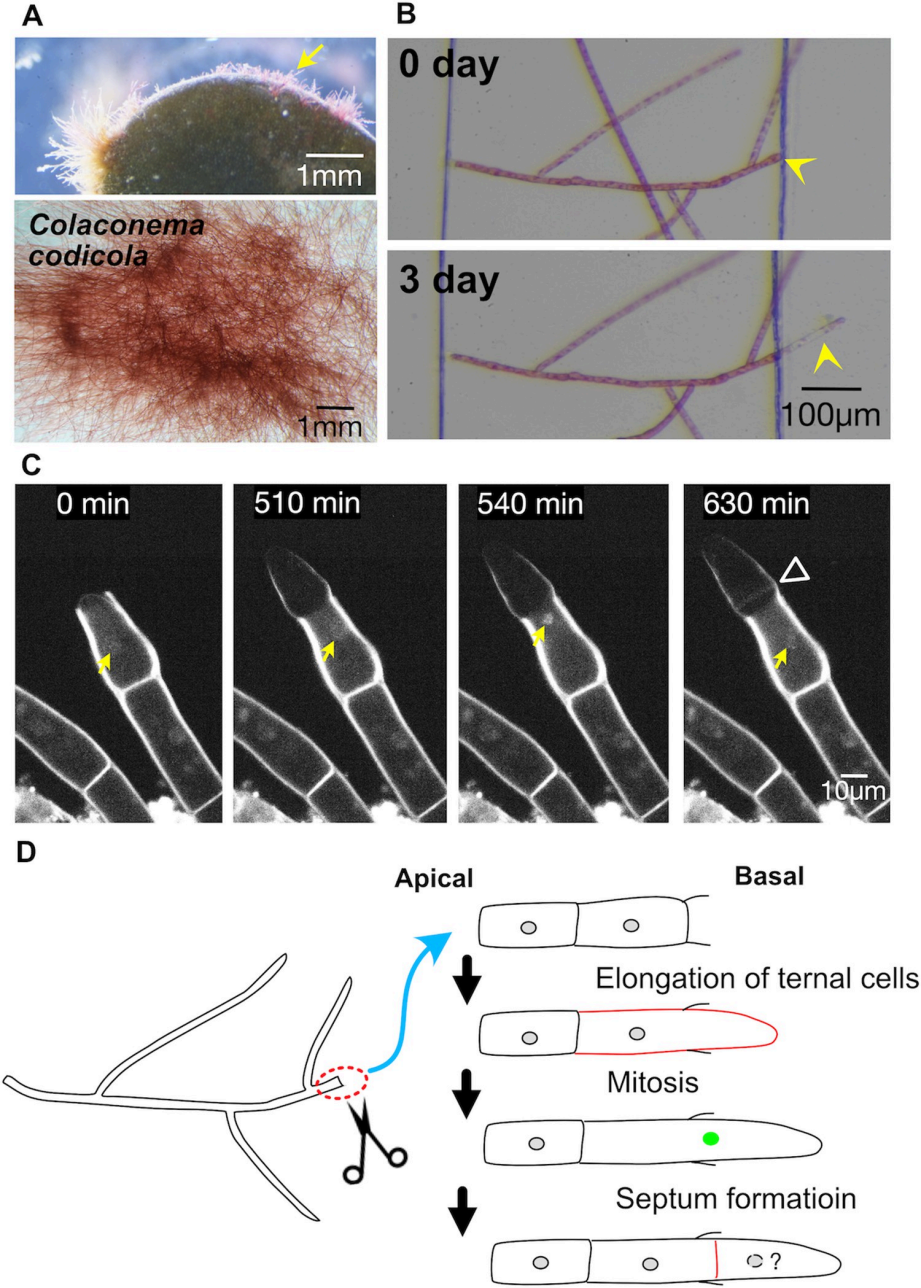
Tip growth, nuclear division, and septation in *Colaconema codicola*. (A) *Colaconema codicola* was isolated from the surface of *Codium fragile* (top). *Colaconema codicola* was successfully cultured in the absence of *Codium fragile* in the laboratory (bottom). (B) Rhizoid formation upon severing. (C) Time-lapse imaging of the nuclei (yellow arrows) of a regenerating cell (rhizoid). The cell next to the injured cell underwent a nuclear division followed by septation (arrowhead) during tip growth. Mitotic chromosome condensation was observed at 540 min, and sister chromatid segregation was complete by 630 min (a sister nucleus is out of focus). (D) Schematic presentation of the injury response in *Colaconema codicola*. Mitotic nuclei are indicated in green.

To elucidate cellular and intracellular responses to injury, four species were studied in more detail. *Colaconema codicola* is a filamentous red alga obtained from the surface of *Codium* (Fig 2A). However, it grew well in seawater without *Codium* in the laboratory. Fig 2B shows rhizoid formation in the basal region after severing the thallus.

Consistent with the DAPI staining of fixed *Colaconema caespitosum* (J. Agardh) Jackelman, Stegenga & J.J. Bolton [26], a single nucleus was visualised in each living cell by Hoechst 33342 dye (Fig 2C). The cell next to the injured site started to regrow (S1 Video). The nucleus was then translocated apically. At 540 min, chromosomes were condensed in a position that was roughly central in the cell, followed by sister chromatid separation and segregation. Septation occurred immediately after chromosome segregation (Fig 2C, arrowhead). Fig 2D schematically describes the injury response.

### Regeneration of multinuclear *Dasya* sp. starts with cell death, followed by neighbour growth, synchronous nuclear division, and septation

**Fig 3:**
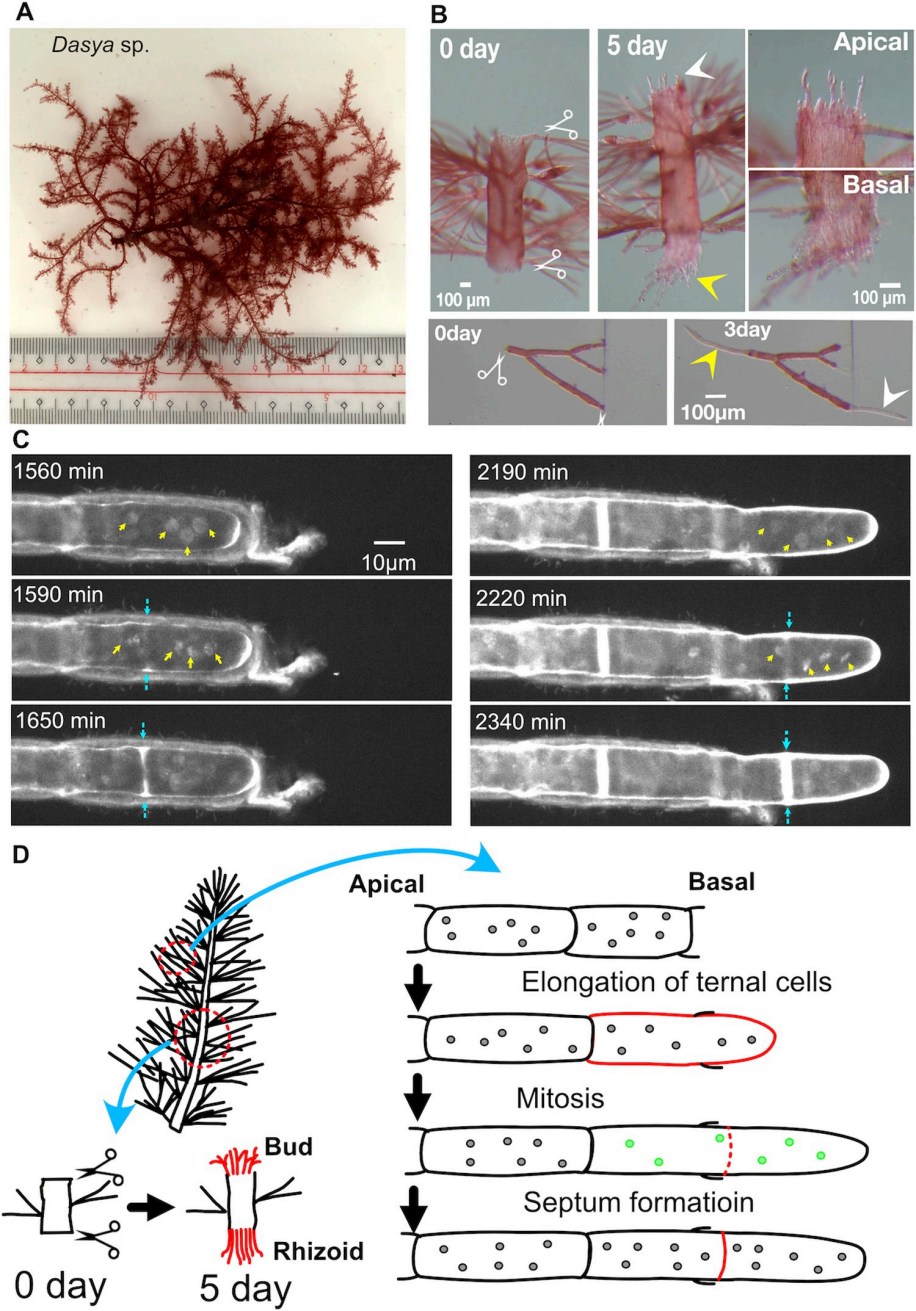
Tip growth, synchronised multinuclear division, and septation in *Dasya* sp. (A) *Dasya* sp. (B) Formation of buds (white arrowheads) and rhizoids (yellow arrowheads) at the apical and basal cut sites, respectively. (C) Time-lapse imaging of the nuclei of a regenerating cell (rhizoid). The cell next to the injured cell underwent two rounds of nuclear divisions and septation during tip growth. Yellow and blue arrows indicate mitotic chromosome condensation and septation, respectively. (D) Schematic presentation of the injury response in *Dasya* sp. Mitotic nuclei are indicated in green.

The red alga *Dasya* sp. develops a cylindrical erect axis, with branches gradually becoming shorter towards the top. Each branch is covered with dense adventitious pseudo-laterals composed of multinucleated cells (Fig 3A). Upon severing of the thallus, new cells were observed in the apical and basal regions and were morphologically indistinguishable. However, only the cells on the apical side formed branches on day 5, indicating that buds and rhizoids formed in the apical and basal regions, respectively (Fig 3B).

In comparison to the clarity obtained with cell wall staining with Hoechst 33342, nuclear staining was not very clear in *Dasya* sp. (Fig 3C, S2 Video). Nevertheless, consistent with a previous description [3], multiple nuclei were observed in each cell (Fig 3C, arrows). The severed cell immediately died (crashed), suggesting that the cell repair mechanisms observed in some coenocytes [9] do not operate in *Dasya* sp. However, the cell adjacent to the injured site started to show tip growth, and concomitantly the nuclei moved in an apical direction (Fig 3C). At 1590 and 2220 min, the chromosomes of all four nuclei in the focal plane were condensed (Fig 3C). The entire process was successfully traced in three cells, all of which showed synchronous mitotic chromosome condensation. Thus, mitosis is likely to be synchronised in *Dasya* sp. The precise timing of chromosome segregation was not visible at this time resolution; nevertheless, it occurred within 30 min after mitotic entry, based on the disappearance of condensed chromosomes. Septation occurred within 1 h of chromosome segregation (Fig 3C, blue arrows). Despite the multiple nuclear segregation events that occurred at one time, the septum was formed only at one site, preserving the multinuclear state of the cell after septation (Fig 3D).

### Regeneration of multinuclear *Cladophora albida* is characterised by simultaneous tip growth of apical and subapical cells, followed by partially synchronous multinuclear division

**Fig 4:**
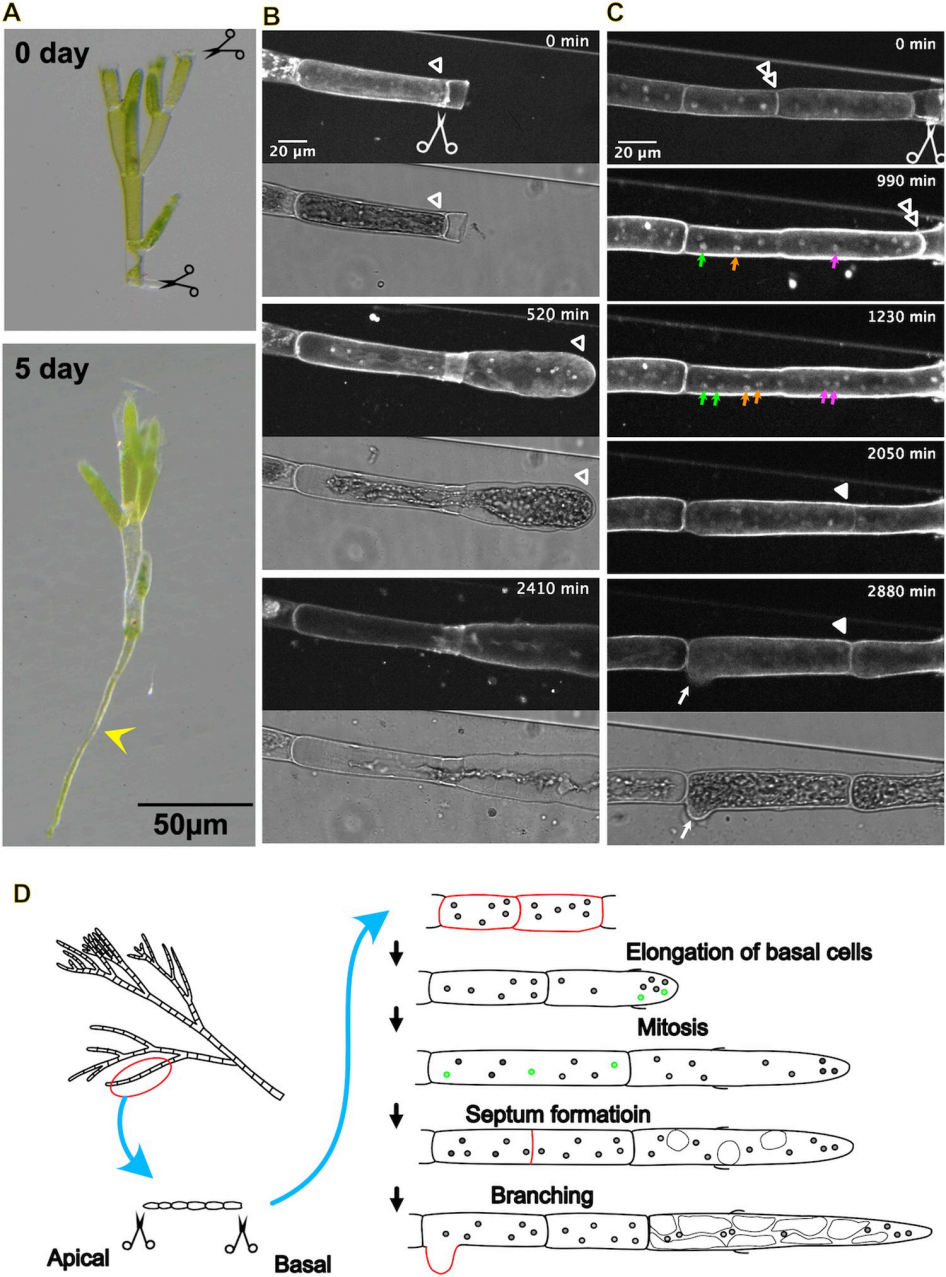
Tip growth, unsynchronised multinuclear division, and septation in *Cladophora albida*. (A) Formation of rhizoids (yellow arrowhead) at the basal cut site. (B, C) Time-lapse imaging of the nuclei of a regenerating cell (rhizoid). The cell next to the injured cell showed tip growth (tips are indicated by open white arrows). (B) In the majority of cases, nuclear division was undetectable, whereas the vacuole developed and occupied the cytoplasm. (C) In other cases, the growing cell underwent nuclear division, not in a synchronous manner, followed by single septation during tip growth. Branching is also observed at the end (white arrow). Green, yellow, and magenta arrows indicate mitotic chromosome condensation, whereas septation is indicated by filled white arrowheads. (D) Schematic presentation of the injury response in *Cladophora albida*. Mitotic nuclei are indicated in green.

The green algae *Cladophora albida* belongs to the family Cladophoraceae. As typical to this family, it develops filamentous, monosiphonous thalli with branches composed of multinucleate cells. Upon severing, all three major responses were observed (Fig 1G [sporulation] and Fig 4A [bud and rhizoid formation]). As previously seen in *Cladophora glomerata* (Linnaeus) Kützing using fixation and DAPI staining [27], multiple nuclei were observed in a cell (Fig 4B–4D). The cell next to the severed site started to regrow rapidly (1.9 ± 0.5 μm/min, n = 14). In addition, adjacent cells further away from the injured site regrew at a slightly slower rate (0.9 ± 0.3 μm/min, n = 9), which was neither observed in *Colaconema codicola* nor *Dasya* sp. In most cases, the cells next to the severed site elongated without nuclear division, and the vacuoles developed rapidly, occupying the cytoplasm in the elongated cells (Fig 4B, S3 Video). In two cases, however, many nuclear division events were observed in the elongated cells (Fig 4C, coloured arrows, and S4 Video), and not all nuclear divisions were entirely synchronised. While synchronous nuclear divisions were observed for some nuclei, others divided at different time points. This is different from that observed in *Dasya* sp., in which all nuclear divisions within a cell were synchronised. Septation occurred infrequently (Fig 4C, white arrowheads).

### Regeneration of *Codium fragile* involves cell repair and new tip growth, followed by nuclear translocation without division

**Fig 5:**
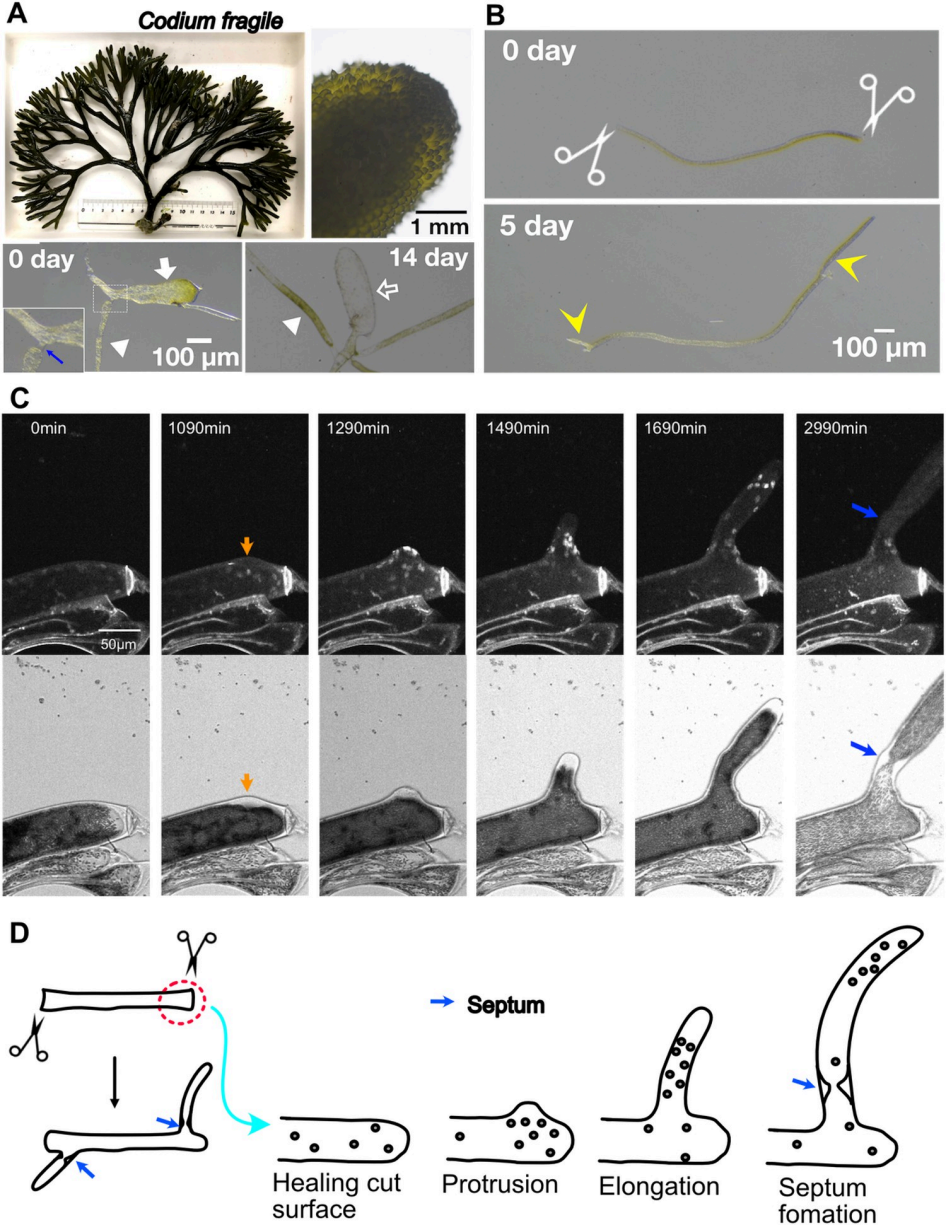
Cell repair, protrusion, tip growth, and nuclear migration in *Codium fragile*. (A) *Codium fragile*. Close-up of the thalli that are composed of closely packed utricles (top right). A single utricle (bottom left, white arrow) with an incomplete septum (blue arrow) and tubular branches (white arrowhead). Fourteen days after isolation, the utricles withered (white open arrow), and the tubular branches grew to form filamentous thalli. (B) Formation of rhizoids (left arrow) and new branches (right arrow) after severing. (C) Time-lapse imaging of nuclei (dyed with Hoechst 33342) (top) and plastids that are visualized by transmission light microscopy (bottom). The cut-repaired site is located on the right (indicated by a strong, linear Hoechst signal). The orange arrow indicates the emergence of a bud. Blue arrows indicate incomplete septation. (D) Schematic presentation of the injury response of *Codium fragile*.

The green algae *Codium fragile* belongs to family Codiaceae, In *Codium fragile*, the thick branches are composed of closely packed utricles, which consist of small cylindrical club-shaped structures (Fig 5A, top right). The structure is characterised by an ellipsoidal portion and two associated medullary filaments (Fig 5A, bottom left). The cytoplasm is continuous, with only an incomplete septum between the filaments and the ellipsoidal portion (Fig 5A, blue arrow) [28, 29]. The structure could be maintained in a petri dish with MSW for less than two weeks; thereafter, the ellipsoidal portion lost the plastids while medullary filaments started to grow (Fig 5A, bottom right). The filament kept growing for more than a year in the petri dish.

Upon severing of a filament at two sites, a unique response was observed, in which the severed site was immediately (< 30 min) repaired or enclosed and growth was observed at both ends (Fig 5B). Hoechst staining confirmed the multinucleate nature of the filaments (Fig 5C and 5D, S5 Video). After 20 h, protrusion and subsequent tip growth were observed adjacent to the cut site (Fig 5C, orange arrow). In contrast to *Dasya* sp. and *Cladophora albida*, mitotic chromosome segregation or chromatid segregation was not observed in the severed area. Interphase nuclei were translocated into the newly formed protrusions without mitosis (1490–1690 min). Once the protrusion grew to a certain length, incomplete septation was observed, through which large organelles such as the nucleus might no longer pass (Fig 5C, blue arrow).

## Discussion

### Macroalgal response to injury

Three injury responses in macroalgae were observed, namely, budding, rhizoid formation, and sporulation. The dominant response depended on the species. Nevertheless, all these responses would be reasonable for macroalgal survival in the natural environment. Rhizoid formation, which often occurred within 3 days, would allow the severed thallus to re-attach to the substrate, while budding efficiently restored the cell types that had been lost by severing. Sporulation occurred within 1 day (*Cladophora albida*) or 14–21 days (*Ulva linza*), enabling the transportation and development of a new thallus.

The ‘negative’ results observed in the assay (either death or no specific response) in 41 samples are not conclusive; the response-ability might be endowed to more species but could not be revealed in the specific experimental conditions in this study (*e*.*g*. lack of water flow, continuous lightening). Furthermore, although sporulation was observed only in green algae in this study, the same response has been detected upon wounding or heat stress in Rhodophyta [30], and this response may be dependent on the age of the thalli. Rhizoid and bud formation occur in the juveniles of some Phaeophyceae species, such as *Sargassum* and *Dictyota* [7, 31], whereas the response could not be observed in the current study for most Phaeophyceae, including *Sargassum*, possibly because mature thalli were used. Thus, the frequency of regenerating species may be underestimated, and this study reinforces the idea that macroalgae have a high regeneration rate [8, 32].

The formation of callus, the cell mass containing undifferentiated cells, could not be observed. The buds formed at the severed site in Rhodophyta *Gelidium elegans* and *Gracilariopsis chorda* were organised in form, thus suggesting the absence of callus formation. Callus formation is the best-known wound-healing response of land plants and has also been observed in many macroalgal species [8, 33, 34]. However, many studies on callus formation in macroalgae are based on *in vitro* tissue cultures, often supplied with extrinsic compounds such as high doses of plant hormones [8]. The observation of the cellular response of four macroalgal species also supports the notion that the callus is not a general intermediate during macroalgal regeneration after injury.

### Injury response at the cellular and intracellular level

This study analysed the nuclear dynamics of macroalgae, based on time-lapse live cell imaging. Generally, fluorescence imaging is challenging for photosynthetic organisms because of the high autofluorescence derived from plastids. Macroalgae are not an exception, and extremely high signals were detected when the samples were imaged with 561 nm and 637 nm lasers, and to a lesser extent, with a 488 nm laser; this last laser is routinely used for GFP imaging. However, autofluorescence was negligible when cells were illuminated with a 405 nm laser, allowing nuclear staining by Hoechst 33342, which is permeable to many, if not all, cell types. Hoechst staining also stained the cell wall, serving as a clear indicator of the cell growth direction and the timing of septation. One caveat of Hoechst imaging is that the low-wavelength laser confers toxicity to cells. In the current experiment, this was minimised by lowering the laser power and exposure time as much as possible and allowing sufficient off-time between image acquisitions. The use of microfluidic devices also made a critical contribution to live imaging, as the nuclear image could be obtained by single focal plane imaging (*i*.*e*. minimal laser exposure). The intact cellular state was very likely maintained during imaging, as evidenced by the occurrence of mitotic cell division (in three species) and nuclear translocation (in *Codium*). Thus, the current method can be applicable to the nuclear imaging of many macroalgal species.

In three species, the injured cells were abandoned irrespective of the number of intact nuclei retained within them, and the neighbouring cells started the reaction. The injury might be recognised by the neighbouring cells, the cytoplasm of which might be connected through pit plugs, a structure formed between adjoining cells during an incomplete septation in cytokinesis in red algae [35]. *Codium* was distinct in that it quickly repaired the injured site, perhaps by membrane closure. The function of the incomplete septum (‘broken wall’) in this process remains unclear. A similar response was observed for *Bryopsis*, which belongs to the same order as *Codium*. A highly viscous cytoplasm might underlie this quick repair of large injuries. The next step after injury was tip growth of the injured or neighbouring cells. Nuclear migration then followed to maintain the overall distribution of the nuclei in a cell. This behaviour is similar to what has been observed in the multinucleated xanthophycean algae *Vaucheria*, where the nuclei are moved towards the branches that have been exposed to blue light [36]. The tip growth and nuclear translocation were commonly observed in all four species. In contrast, there appeared to be a substantial variation in cell cycle control. While *Dasya* cells underwent synchronous nuclear division, this was not the case in *Cladophora*. In *Cladophora*, the nuclei that were closely located to each other tended to undergo synchronous division, whereas those in other areas did not, despite sharing the same cytoplasm. *Codium* is extremely different, in that mitosis has been scarcely observed during regeneration. In > 10 imaging attempts, only one nuclear division was observed among > 100 nuclei. The injury response of *Codium* may represent cellular wound healing, which does not affect cell cycle control. Finally, the formation of the septum, including the incomplete septum in *Codium*, was a variable process. In Rhodophyta *Colaconema* and *Dasya*, this was tightly coupled with nuclear division and formed immediately after nuclear segregation. In contrast, septum formation was loosely coupled with nuclear division in (multinucleated) Chlorophyta, *Cladophola*, and *Codium*. In the multinucleated fungus *Aspergillus nidulans* G Winter, septation occurs once the tip has grown to a certain length [37]. However, this does not appear to hold true for *Cladophora*, as it could not be inferred whether and when septation takes place based on cell length (Fig 4B and 4C). The regulation of septum formation is an interesting topic for future research.

In conclusion, the collective findings shed light on the common features as well as the various pathways of regeneration in marine macroalgae. Four highly-regenerative species amenable to live fluorescence microscopy are of particular interest, as they have a potential to be the model macroalgal species for experimental cell and developmental biology.

## Supporting information

S1 Fig Macroalgae collection sites. (A–C) The three sites of macroalgae collection. Outdoor tank at the Sugashima Marine Biological Laboratory (NU-MBL) which had a continuous flow of unfiltered seawater (A), the underwater rope at the pier to which macroalgae were attached (B), and the intertidal zone in front of the NU-MBL (C).
(TIF)

S2 Fig Microfluidic device for live-cell imaging of macroalgae. (Left) A PDMS device, 15 μm in height, was attached to the glass-bottom dish. (Right) Severed thalli of *Cladophora albida* were injected into the device.
(TIF)

S3 Fig Examples of budding and rhizoid formation after injury in red macroalgae. Buds (white arrows), rhizoids (yellow arrows), and severed sites are indicated.
(TIF)

S4 Fig No response to injury in some red macroalgae species. (TIF)

S5 Fig No response to injury in some red macroalgae species. (TIF)

S6 Fig Rhizoid formation after injury in a brown algae, (*Feldmannia mitchelliae*). Rhizoids (yellow arrows) and severed sites are indicated.
(TIF)

S7 Fig Examples of rhizoid formation and sporulation after injury in green macroalgae. Rhizoids (yellow arrows) and severed sites are indicated.
(TIF)

S8 Fig No response to injury in many brown macroalgae species. (TIF)

S9 Fig Budding at the severed surface in *Gelidium elegans*. Stereomicroscopic images of the severed surface of *G*. *elegans* thalli at day 0 (immediately after severing), 3, and 7. (B) Frozen section images of a cut surface of *G*. *chorda* thalli. Inset: enlarged image.
(TIF)

S1 Video Tip growth, nuclear division, and septation in *Colaconema codicola*. Time-lapse imaging of Hoechst 33342 using spinning-disc confocal microscopy. Arrow indicates condensed chromosomes in mitosis.
(AVI)

S2 Video Tip growth, synchronised multinuclear division, and septation in *Dasya* sp. Time-lapse imaging of Hoechst 33342 using spinning-disc confocal microscopy. Arrows indicate condensed chromosomes in mitosis.
(AVI)

S3 Video Tip growth without nuclear division or septation in *Cladophora albida*. Time-lapse imaging of Hoechst 33342 using spinning-disc confocal microscopy.
(AVI)

S4 Video Tip growth, unsynchronised multinuclear division, and septation in *Cladophora albida*. Time-lapse imaging of Hoechst 33342 using spinning-disc confocal microscopy. Arrows indicate condensed chromosomes in mitosis.
(AVI)

S5 Video Tip growth, nuclear migration and incomplete septum formation in *Codium fragile*. Time-lapse imaging of Hoechst 33342 using spinning-disc confocal microscopy.
(AVI)

S1 Table DNA barcode sequences of several macroalgal species. (XLSX)
