# Supplementary figures and images for: Cell tip growth underlies injury response of marine macroalgae
Source: PLoS One. 2022 Mar 17;17(3):e0264827. doi: 10.1371/journal.pone.0264827 (PMC8929694; doi:10.1371/journal.pone.0264827)

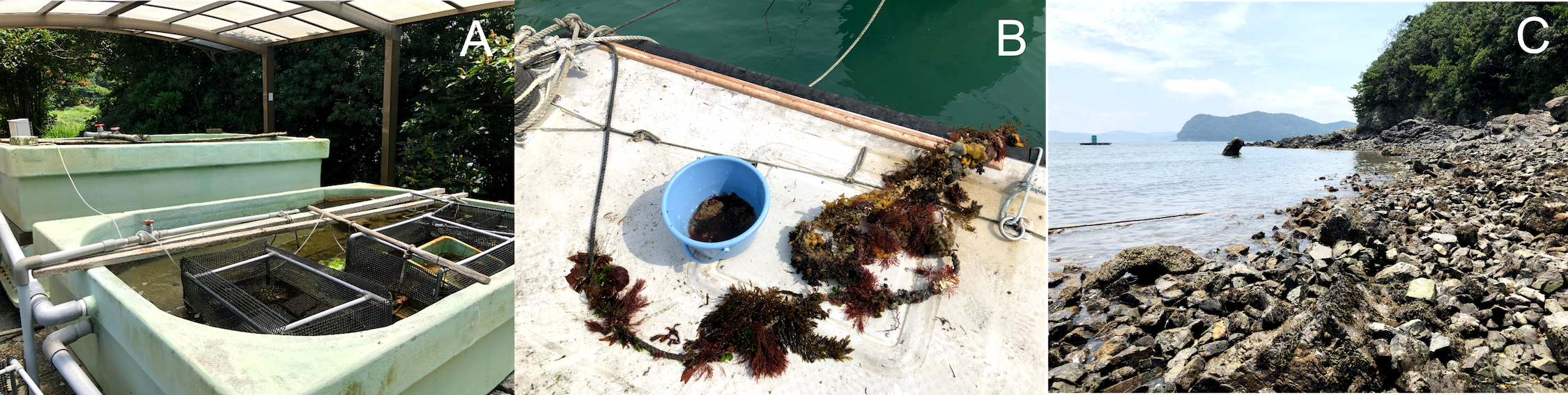

Supplement: S1 Fig — (A–C) The three sites of macroalgae collection. Outdoor tank at the Sugashima Marine Biological Laboratory (NU-MBL) which had a continuous flow of unfiltered seawater (A), the underwater rope at the pier to which macroalgae were attached (B), and the intertidal zone in front of the NU-MBL (C). (TIF) [file pone.0264827.s001.tif]

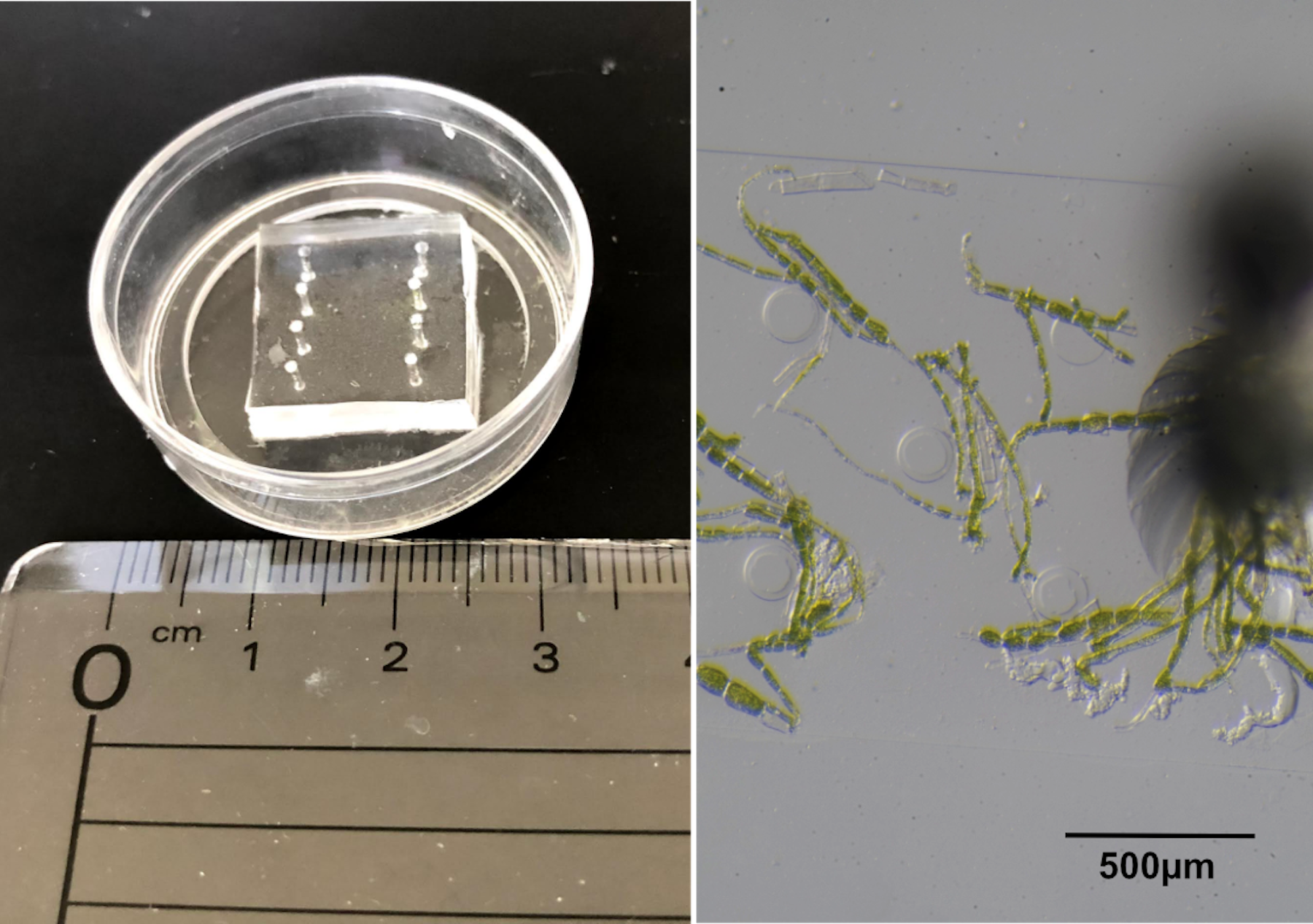

Supplement: S2 Fig — (Left) A PDMS device, 15 μm in height, was attached to the glass-bottom dish. (Right) Severed thalli of Cladophora albida were injected into the device. (TIF) [file pone.0264827.s002.tif]

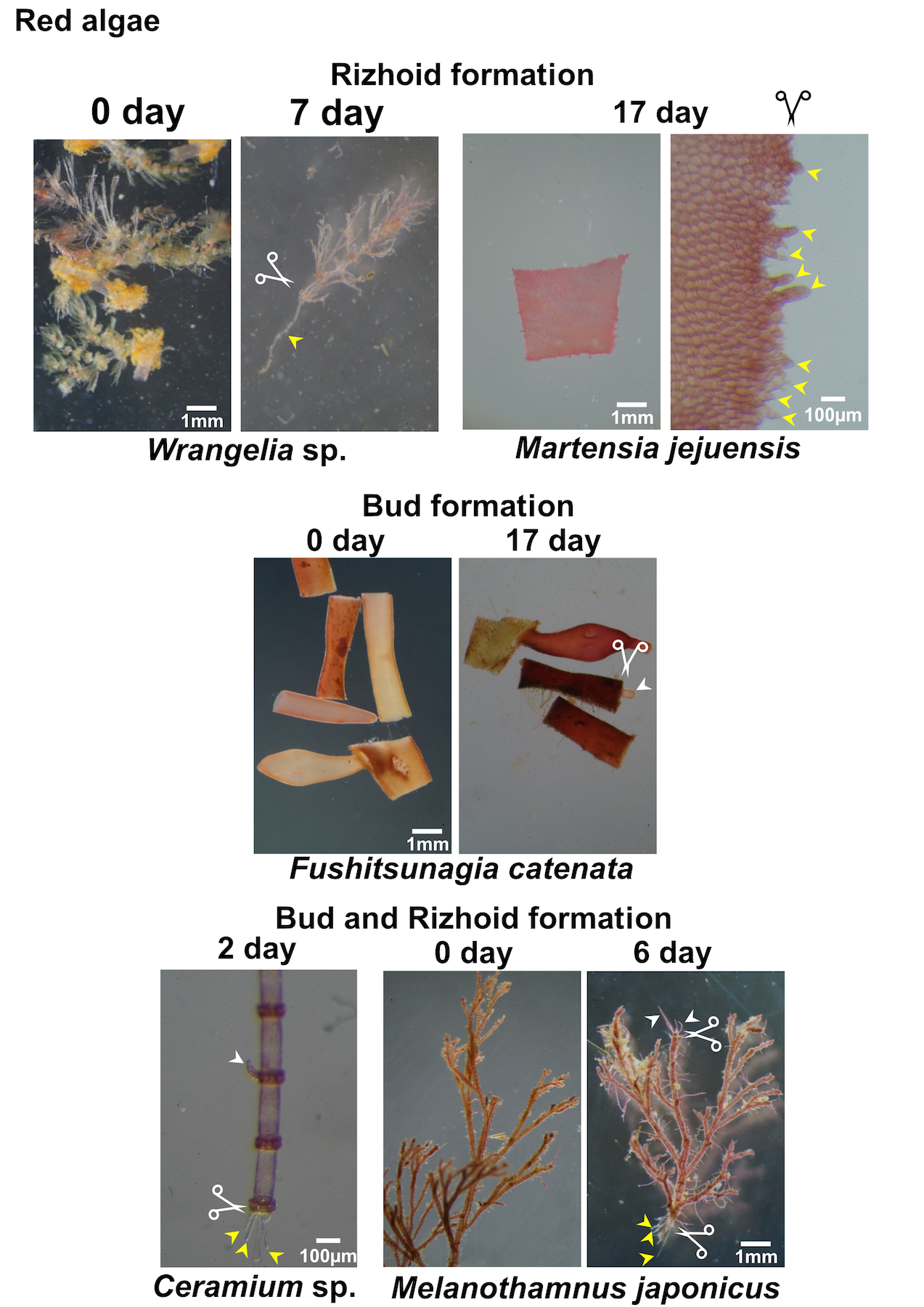

Supplement: S3 Fig — Buds (white arrows), rhizoids (yellow arrows), and severed sites are indicated. (TIF) [file pone.0264827.s003.tif]

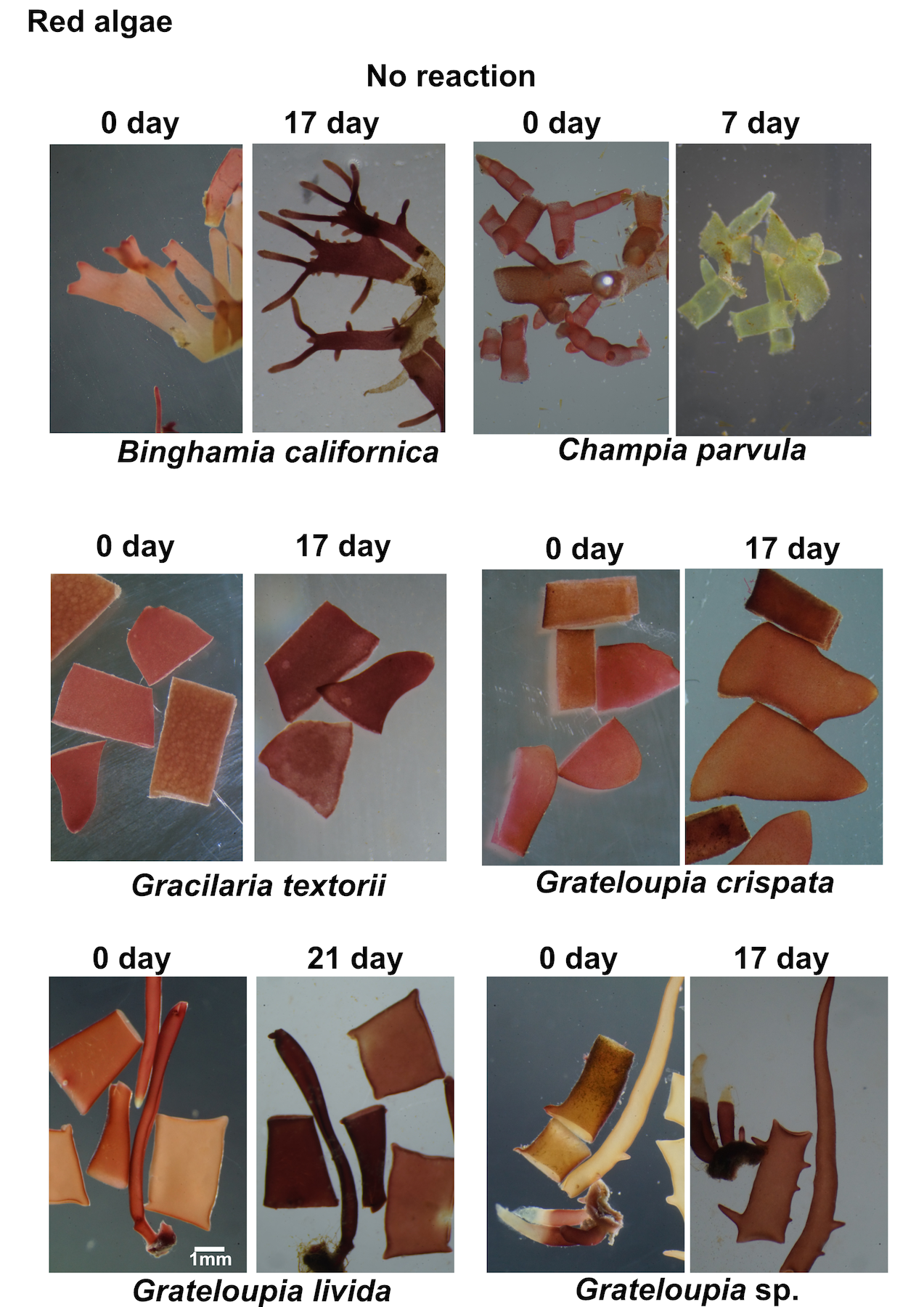

Supplement: S4 Fig — (TIF) [file pone.0264827.s004.tif]

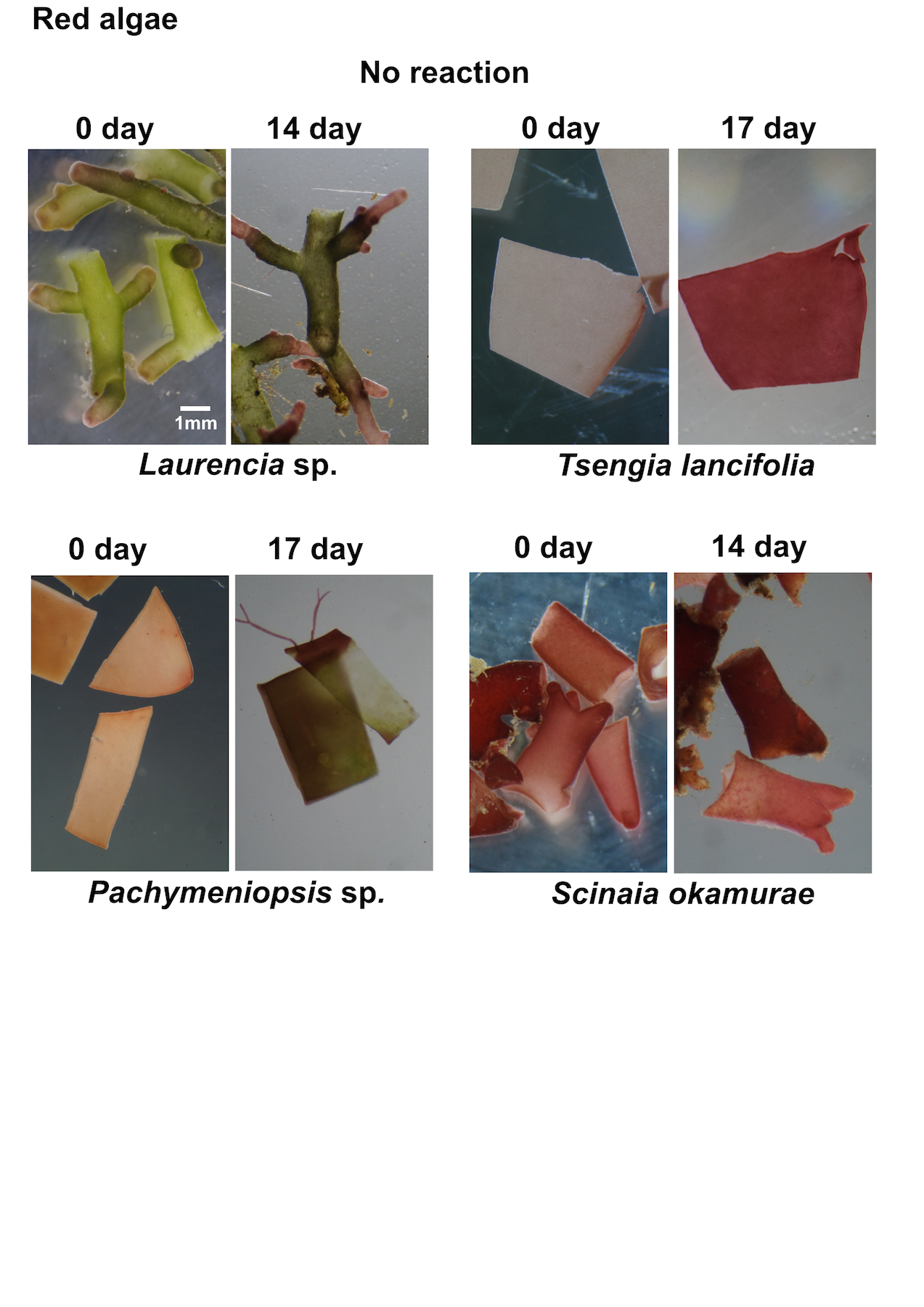

Supplement: S5 Fig — (TIF) [file pone.0264827.s005.tif]

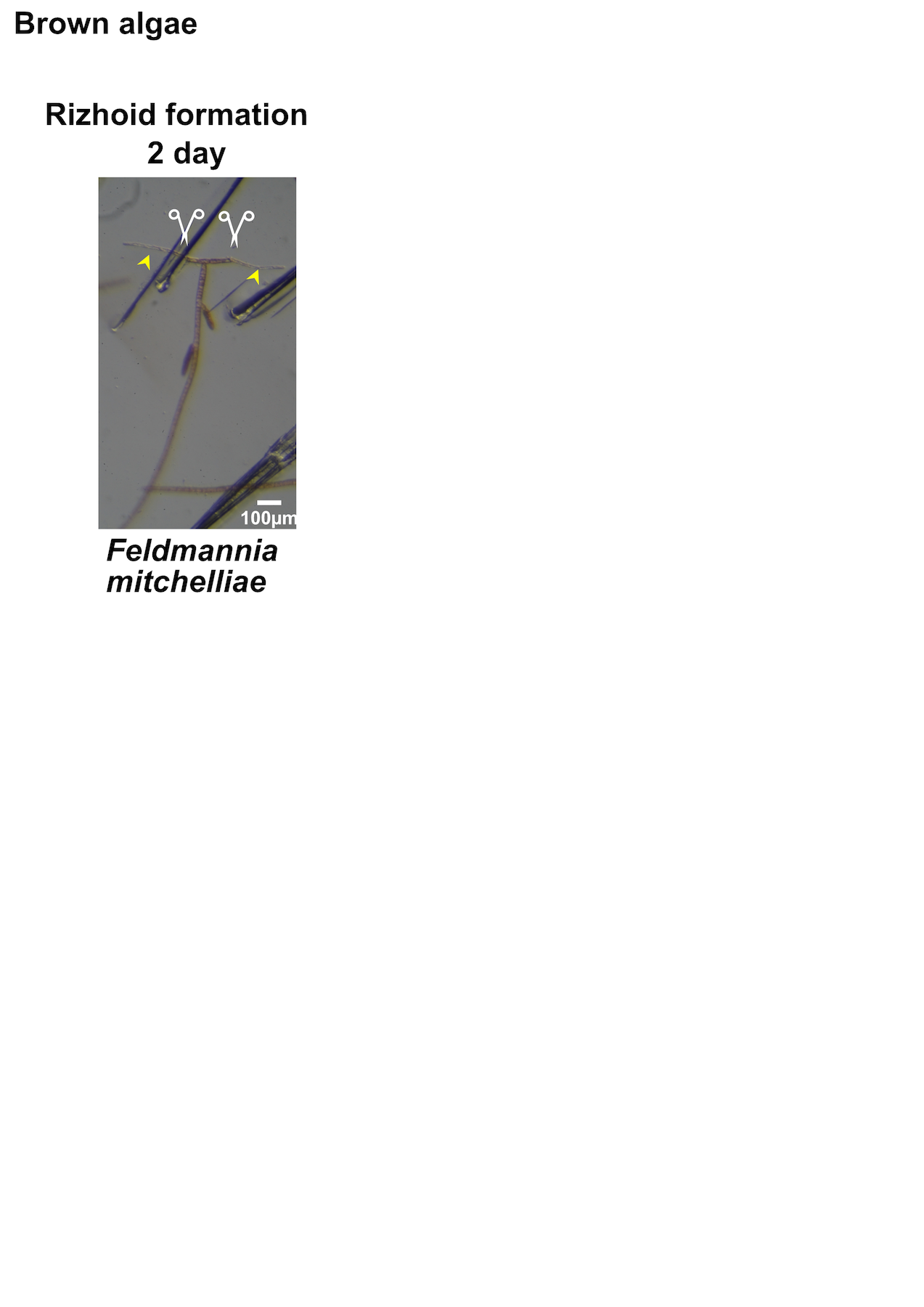

Supplement: S6 Fig — Rhizoids (yellow arrows) and severed sites are indicated. (TIF) [file pone.0264827.s006.tif]

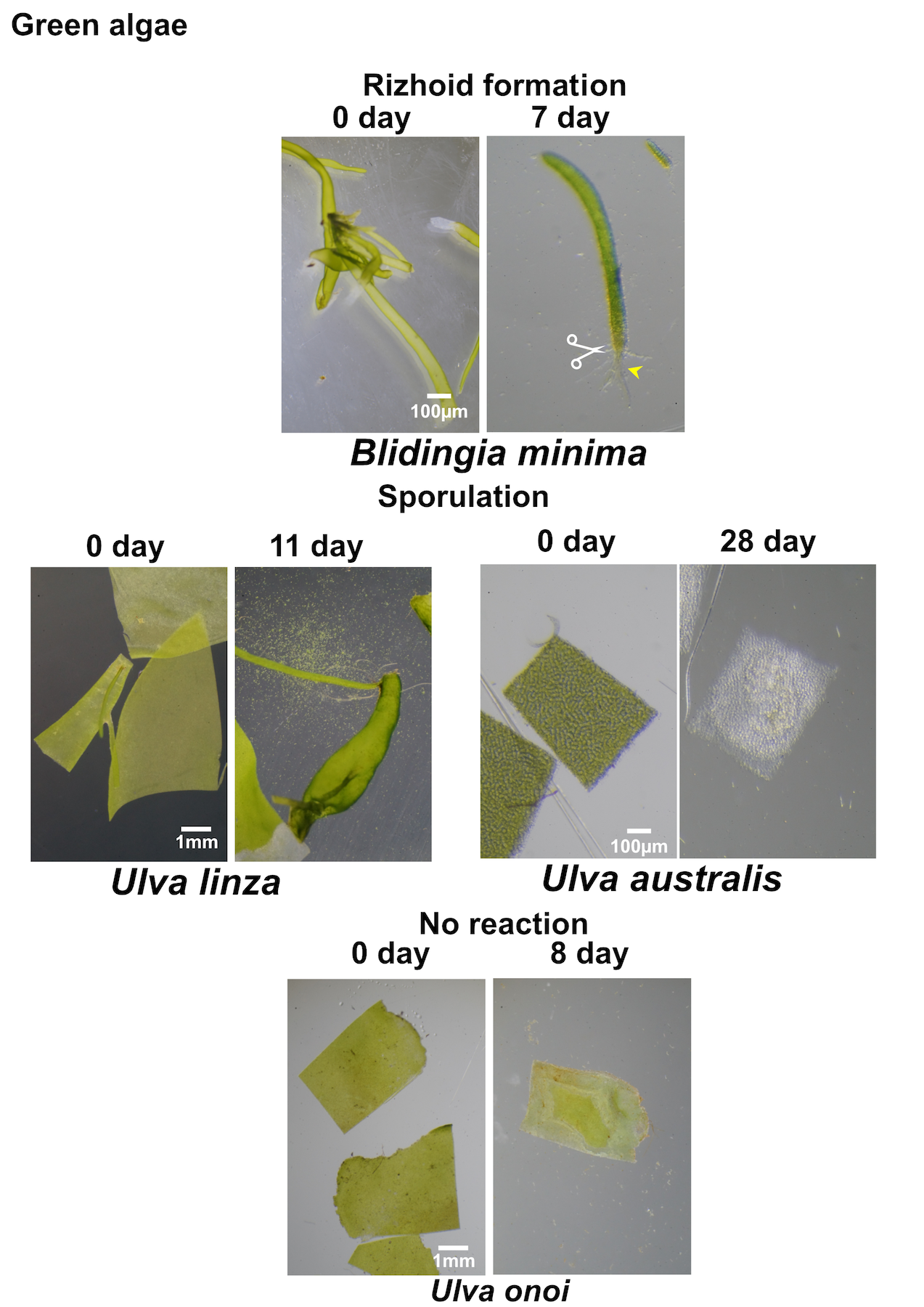

Supplement: S7 Fig — Rhizoids (yellow arrows) and severed sites are indicated. (TIF) [file pone.0264827.s007.tif]

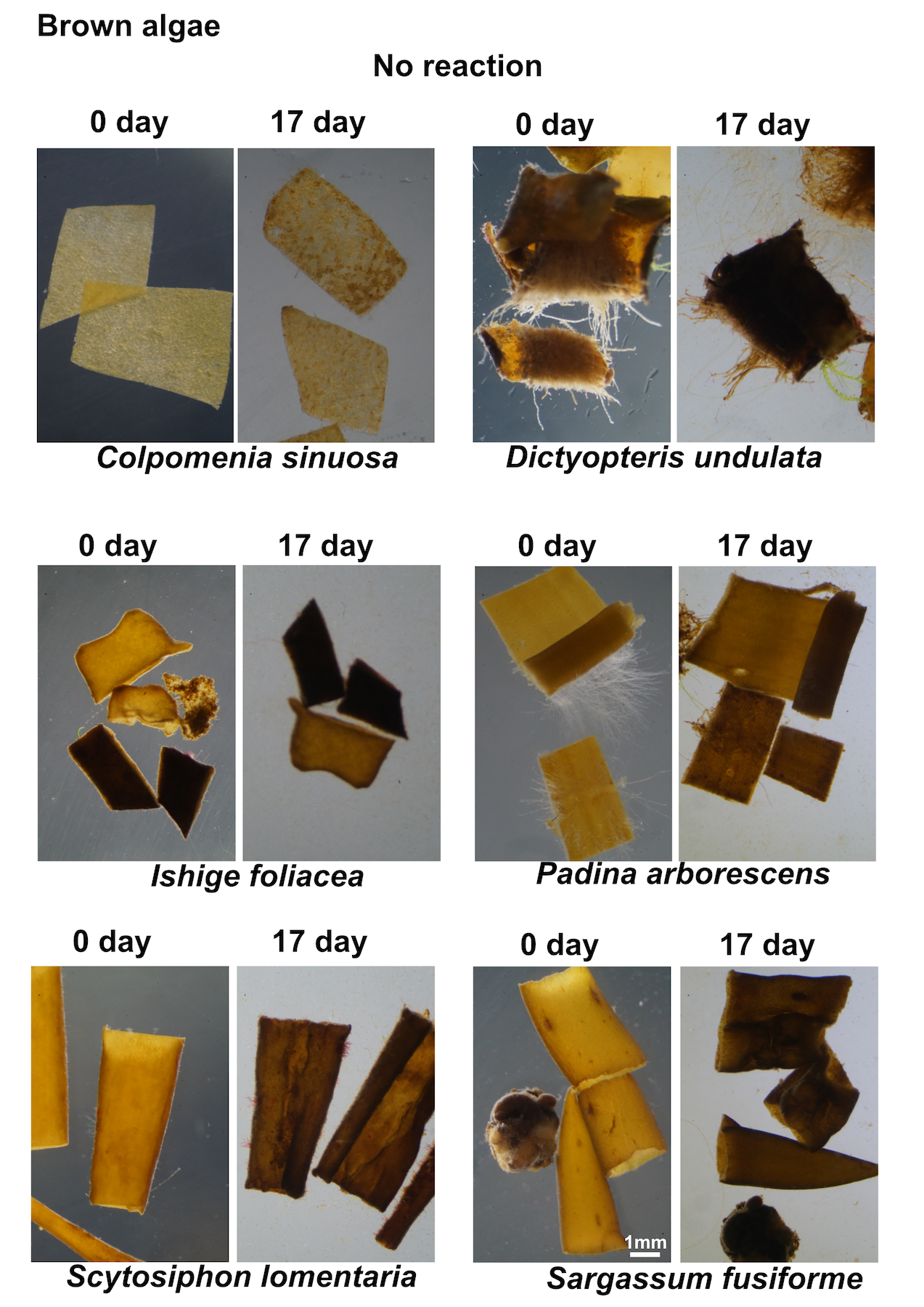

Supplement: S8 Fig — (TIF) [file pone.0264827.s008.tif]

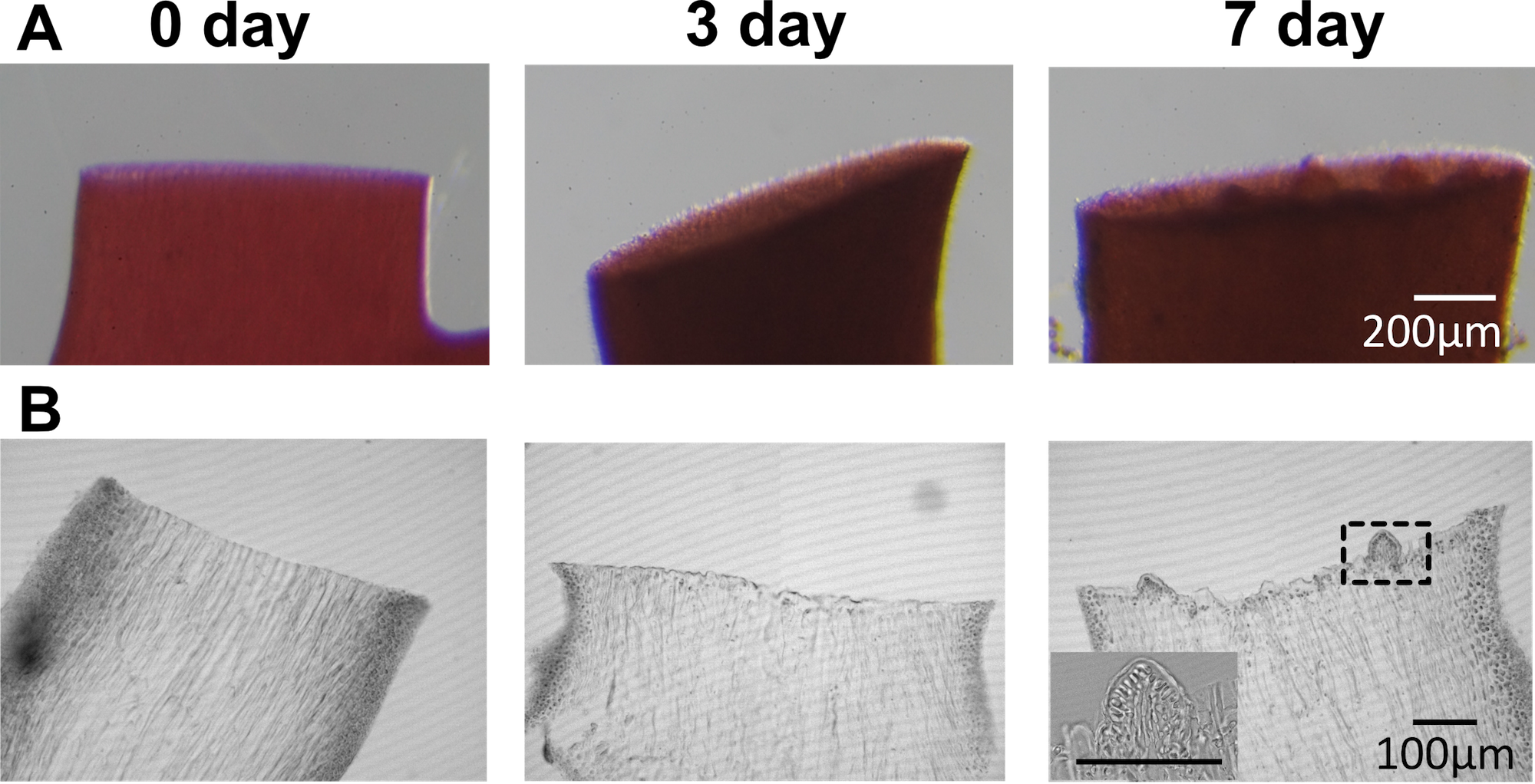

Supplement: S9 Fig — Stereomicroscopic images of the severed surface of G. elegans thalli at day 0 (immediately after severing), 3, and 7. (B) Frozen section images of a cut surface of G. chorda thalli. Inset: enlarged image. (TIF) [file pone.0264827.s009.tif]
